# Mr. Scott, on Hæmatocele

**Published:** 1809-05

**Authors:** James Scott

**Affiliations:** Surgeon. H. M. S. Euryalus, Sheerness


### *To the Editors of the Medical and Physical Journal.*

Gentlemen,

On a former occasion,* you did me the favour to publish my Observations on a case of Scrotal Hernia, which as they stated an opinion at variance with the practice of one of our best modern surgical writers, may have been thought rather novel than practically useful; but, as mere accident has frequently led to the greatest improvements in science, it becomes the duty of every medical man faithfully to record unexpected occurrences in disease, which may often furnish the ingenious philosopher with hints very beneficial to mankind.

On the subject of my last paper, authors are divided, and we find one, not only treating wounds of the septum scroti as of no consequence, but he even gives a case wherein it was intentionally perforated by himself.

This circumstance, coming from such authority, certainly speaks against my theory, yet, I trust I may be allowed to suppose, that in Mr. Latta's case, the septum may not have been very vascular; or it is surely possible, and, I think, highly probable, that the instrument passed between the vessels, particularly as Mr. Latta says, that he divided the part by cautious scratches.

If, however, it be allowed that hæmatocele has very suddenly followed the palliative operation for hydrocele, as we are informed by most respectable writers, then I am persuaded the locality, which the septum may occasionally assume, will sufficiently account for the production of that disease.

 In

* See Medical and Physical Journal, No. 99, p. 430, May, 1807.

In my former paper on this subject, I did admit, merely to avoid unprofitable controversy, that the vessels might perhaps burst when the support of the serous fluid was removed; but such an explanation was always very unsatisfactory to my mind, and I now utterly discredit it.

The hæmatocele has been defined a collection of blood within the vaginal coat of the testicle; and it would appear, by the language of authors, to be the consequence of a rupture of the vessels of that part; but our anatomical knowledge is unfriendly to such a supposition, as we are not thereby made acquainted with any vessels which being wounded, could induce such a complaint; and how far the evacuation of a morbid fluid may operate in this way, is at best problematical, since analogy surely does not furnish us any ground to stand on.

Ophthalmia is the species of inflammation, on whose symptoms and consequences we ought chiefly to reason, because they are most obvious to our senses; and as natural causes and effects are known to operate by general, and not by partial laws, we have a right to infer, that inflammation generally commences and proceeds in the same manner that inflammation of the eye does. The parts most generally affected with this complaint, are similar in structure to the tunica vaginalis and albuginea testis, in as much as they are all nourished by blood-vessels which carry no red globules, and whose surfaces are uniformly lubricated by a limpid fluid. In ophthalmia, a superabundant quantity of tears is secreted by the lachrymal gland, in consequence of the irritating cause, whatever it may be, that induces the disease; and the same stimulus also occasions a defluxion of serum from the vessels of the eye; but in no instance, I believe, has it ever happened that a hæmorrhage took place from the red vessels on the sclerotic, except it was solicited by careful scarification of the part, and even then practitioners have cause to lament, that only a very small quantity of blood can be obtained by such an operation.

In the testicle, the exhalents of the tunicæ vaginalis and albuginea, furnish a fluid similar in use to the secretion of the lachrymal gland; and, when the absorbents have their functions impaired or destroyed by external or other causes, connected sometimes with general disease, a morbid accumulation of fluid, or hydrocele, is the consequence. This complaint is extremely frequent, and we are told by authors, that puncture of the scrotum, to relieve it, has been productive of a disease much more serious in its nature, viz. hæmatocele; and further, that all attempts attempts to palliate or remove this collection of blood, have only hastened the destruction of the patient.

If this statement is correct, to what source shall we trace the flow of blood? Can we on mature consideration believe that vessels, so strong as those which supply the coats of the testicle, and naturally carry no red blood, can burst merely because the pretended support of a morbid fluid is removed? or that, supposing them to be ruptured by such a cause, or by other means, they could furnish several pounds of pure blood? Our reason revolts at such an explanation of a fact, which all surgeons, who have seen many cases of hydrocele punctured, must have witnessed in some degree.

I will not deny, that very often the trocar may wound a vein, or an arterial branch of the scrotum, and cause a little blood to be poured out when the canula is withdrawn; but such is the contractile nature of the scrotum, that no hæmorrhage of consequence can happen in that manner; and if a vessel were divided, it would certainly in a few minutes retract into the cellular substance, and the bleeding must soon cease.

But it is much easier, and, in my opinion, much more rational to suppose, that a wound of the vessels of the septum scroti occasions hæmatocele, both on account of the reasons advanced above, and because the wound of the scrotum, if inflicted by a lancet-pointed trocar, is commonly healed in a few hours; in addition to which may be mentioned the more favourable situation of the vessels of the septum for active hæmorrhage, in consequence of their seclusion from the influence of atmospheric air, and, of course, their equable temperature, which by preserving the blood in its natural state of fluidity for a short time, would procure it an easy introduction to the cavity of the vaginal coat.

Should any of my medical Brethren, whose long experience may have afforded them opportunities of forming a more accurate conception of the disease than I have done, be inclined to favour me with their opinion, through the medium of your Journal, it would afford me much satisfaction; and, should I even discover, by liberal discussion, that I have entertained a wrong notion of the complaint in question, I shall at least have the pleasure of reflecting, that I was actuated by no motive but an ardent desire to be useful in the profession I have chosen, and to assist in smoothing the rugged path of humanity.

JAMES SCOTT, Surgeon.

*H. M. S. Euryalus,*

